# The individualized supervision strategy and effectiveness under the strength perspective: a pilot study for the case management model of the high-care elderly in communities

**DOI:** 10.1186/s12913-021-06574-2

**Published:** 2021-06-04

**Authors:** Yu-Ming Chen, Kuei-Min Chen, Chiang-Ching Chang, Meng-Chin Chen, Li-Yen Yang

**Affiliations:** 1 National Penghu University of Science and Technology General Education Center, Penghu, Taiwan; 2 Kaohsiung Medical University College of Nursing, and Kaohsiung Medical University Hospital Department of Medical Research, Kaohsiung, Taiwan; 3 Department of Medical Sociology and Social Work, Kaohsiung Medical University, Kaohsiung, Taiwan; 4 Department of Nursing, Yuhing Junior College of Health Care and Management, Kaohsiung Medical University College of Nursing, Kaohsiung, Taiwan; 5 Fooyin University College of Nursing, Kaohsiung, Taiwan

## Abstract

**Background:**

This study tried to improve the operational efficiency and service quality of the community case management model for the High-Care Elderly in Communities in Taiwan. This study tried to assistant social workers in community care sites to establish individualized treatment planning, to empower them with flexibility to discover and utilize their own unique strengths, to reach the goal of developing community care capacity.

**Methods:**

A case consulting model was employed in this study by providing case by case supervision service. We collected all supervision meeting records and related data as our qualitative research materials, analyzed these materials and tried to define intervention effectiveness of the individualized supervision strategy under the strength perspective applied to the case management model of the high-care elderly in communities. To find out what helps these social workers the most, and how these social workers realize and recognize their process and key to improve their service quality and work efficiency, in the way of qualitative interpretation.

**Results:**

A supervision model under the perspective of strength was developed in this study, and was applied to clinical community care sites to help their social workers. The results proved that our supervision model provided an opportunity to empower and re-know themselves, re-establish their professional confidence and meaning of existence for their organization, and eventually produced positive effect to quality of service and service receiver’s satisfaction improvement. The key feature that made this supervision model of the strength perspective work was to let social workers have the autonomy to interpret the challenges they face and to think possible alternative solutions creatively.

**Conclusions:**

This study showed that create a supervisee-friendly psycho-socio environment empowered these workers by increasing their resource network and taking advantage of what they still have and what they were good at, this could help these workers upgrade their level on ladders of empowerment, become an active and independent professional worker that have power and confidence to make treatment and intervention.

## Introduction

Nowadays, Taiwan have become an aged society. The percentage of people aged over 65 was 7% in 1993, increasing to 14% in 2018, resulting in more people aged over 65 than those under age 15. The working population is decreasing and caregiving human resources are lacking, indicating that we have to be prepared and improve service quality and effectiveness in long-term care for the aging population in the future [1]. Meanwhile, the trend of increasing average expecting life length not just improved aged population’s well-being and quality of life, but also brought some problems into the society. For example, average expecting life length was already over 80 years for Taiwanese people since 2016, but average expecting healthy life length was only about 71 years, suggesting that most aged people in Taiwan have to experience about 9 to 10 years under the situation of sub health or with disability requiring long-term care. This situation undoubtedly increased burden of life and caring for family caregivers, highly increased the risks for an aged person become a potential high-care case. In addition, it also made the existed urban-rural gap issues in care resource inequity become more serious [2]. Another related problem in Taiwan was that the traditional cultural custom and Confucian family ethics made most Taiwanese aged people tend to live with their children and to regard taking care for them as duty and obligation of their children, so when some aged people have needs for long-term care service or face high risk situation, they and their children often feel afraid, guilty or ashamed and suffer from social pressure, to be a unqualified and defective children to seek for asking professional aid from outside. Therefore, we thought that the importance of find out potential high-care elderly and strengthen capacity of primary community care should be highly valued to meet the 5As of “Principle of social resource operation”: accessibility, acceptability, availability, affordability, accountability for long-term care services [3].

Long-term care policy model of Taiwan could track back to “Long-Term Care ten-year program” since 2008, and renewed as “Long-Term Care 2.0” since 2017 as mainstream official model for promoting community care and home care to meet the key concept: aging in the place, to let the elderly could enjoy their later life in a familiar living environment and social network. Therefore, to develop and empower capacity of primary community care and local care giving human resources, become the key issue of Taiwanese Long-term Care policy practice. Now in primary community care practice, most local basic caring services and social work intervention delivered by C level Community Care Site of “Long-Term Care 2.0”, that mostly held by local non-profit organizations or volunteer groups, included usual visiting and telephone call caring, consultation and referral services, catering services and health promotion activities, to implement the function of health promotion and primary prevention of community care. One of the key problems of this model, is that Taiwanese LTC policy model were deeply influenced by Welfare Pluralism, that made the government would just sponsor the budget of community care site, or any other social service deliver NPO in a low rate. Since that, many community care sites always face difficulties of regular budge and professional human resources shortage; Hsiao [4] pointed out that there were challenges such as over-loading of the community, democratic accountability and new managementism, hardware subsidy tightening and insufficient professional counseling. As a result, the proportion of personnel costs in the operation of community care sites become unaffordable, the cases were easily lost, then made the community care sites could not be sustainable.

The challenges mentioned above actually reflected problems of insufficient professional manpower and insufficient follow-up case management skills [5]. So there was another problem that influence service quality of community care sites, because the correctness of valuation of high-care elderly cases depend on social worker’s experience and professional abilities, since these community care sites often lack of sufficient professional social workers, how we could expect them to find out those elderly, who indeed need intervention and LTC services [6]?

Through the above analysis of the current situation and possible problems, and challenges of Taiwan’s high-care elderly service intervention model, since it’s hard to change the operation model and budge rules of government LTC policies and programs immediately, we authors as a team of LTC teachers and researchers tried to solve or ease the problem in the other creative way: improve the operational efficiency and service quality of the community case management model, and assistant those social workers in local community care sites by establishing individualized treatment planning that was different from the mainstream model, empower them with flexibility to take advantage of their own unique strengths, to reach the goal of developing community care capacity, then interview and analyze those who experience new supervision model about their comments and feelings to make practical suggestions to improve LTC policies and clinical supervision programs through this study.

## Method

### Theoretical framework

In this study, the perspective of interpretivism was employed through in-depth interview and narrative analysis methods to understand how those social workers in the community care sites get help using their unique strengths and their subjective interpretation from the new integrated high-care case management model with a five-stage care delivery model was formulated: case screening, case assessment, care plan, care delivery, and follow-up evaluation, and classify six types of high-need older adults were identified: those living with disability, solitude, dementia, depression, elder abuse, and poverty with an integrated list of categorized care services, including care resources, social welfare resources, and caregiver resources, was then used as a guide for case management and care delivery, that help workers working more efficiently and rapidly [7].

### Sampling

In order to find representative samples in Taiwan, a case consulting model was employed in this study by providing case by case supervision service [5] to encourage the social workers in community care sites to invent resources for the unit, to analyze advanced clinical case intervention planning and to conduct follow-up evaluation.

All recruited volunteering social workers would be given consultations every 3-4 weeks for 18 months. One group supervision meeting, led by an experienced and certified expert in social work in our research team, was provided for all sample sites every 3 months. The focuses of the group supervision meetings were: A. Difficulties of a local community care unit that face the national and general expects from official long-term care policies and the public; B. How to adjust and balance operation policies while social, material and human resource lacking; C. How to create their own distinguishing features and advantages; D. Strategies to “work with” but not “work for” the underprivileged clients to exert their strength that they still have.

### Research design

To lower social worker’s loading and burden through the perspective of strength by our consultation, tried to help them analyze and understand their (personnel for the social worker or structurally for the organization or the community) service potential and strength for clients, either in qualitative or quantitative data, designed service resource handbook for every unique site, empowered these social worker by service experience sharing, peer group support, group brainstorming, to open their vision and rich their service options, to realized their expecting mission: primary care and health promotion in community, and eventually face-to-face interviewed 8 social workers in their meeting room or counseling room from 4 different community care sites in both rural and urban areas of Kaohsiung city in southern Taiwan, exclusion criteria was to exclude those community care sites workers that were not social worker, by purposive sampling.

### Analysis method

By this research design, we collected all supervision meeting records and related data as our qualitative research materials, and employed open coding to classify the interview data from three aspects according to the result of the literature review and our observation in the field: A. How these sites try to establish their own high-care elderly case management model to help clients and what are their expectations to the supervisors; B. How to realize the development of strategic recommendations; C. How the strength perspective be helpful for community social workers; to find out the common points of the effective factors of the model from the various records of the interviewees, and analyzed those classic samples’ comments and content of transcripts of their descriptions to illustrate how them be help in this intervention model and discussed more how could we improve the supervision model together or re-correct our analysis and interpretation, if necessary arrange more times of interview till data saturation, by analyzed those records that all samples mentioned of in common. In order to define intervention effectiveness of the individualized supervision strategy and effect under the strength perspective that applied to the case management model of the high-care elderly in communities, and how these social workers explain and recognize their process and key to improve their service quality and work efficiency in the way of qualitative interpretation, to understand the reasons for the gap between the deductive and practical operations of the theories [8], to explore possible practical improvement strategies under Taiwanese local social context. Therefore, our semi-structured interview guide was designed as below:

1. What is the current operating model and expectation of the government of your community care unit?
2. What make gaps between the current operating model and the supervision model of your community care unit? What are the specific difficulties?
3. Please make comments for the influence of the new individualized supervision strategy and effectiveness.

All methods were carried out in accordance with relevant guidelines and regulations. And informed consent was obtained from all subjects.

## Result

After interview data collecting, the first, third author and one research assistant coded the data together, and analyzed it by the authors under the perspective of strengths and applied to the context of practical LTC service by the following aspects to classify our research finding.

### The establishment of high-care elderly case management supervision mode and the development of strategic recommendations

Now in the most community care sites of Taiwan, unstable, mobile, less-experienced human resource issue is a big problem that makes these workers could not receive deeper training, continue accumulating experience, and then easily become an unconfident, frustrated, low self-respect worker that cannot devote enough time and effort in the community [9–11]. These issues make the community care sites could not realize their expecting function of high-care elderly caring and socio-psycho problem intervention; but due to there were various kinds of community care site, every site has their own unique specialty and characteristics to grow their service capacity, so we knew that we have to discuss a supervision model that is flexible and could adapted to every site’s characteristics and unique specialty: the strength perspective that encourage NPOs and their workers to develop their own special and unique comparative advantage and strength [12], that is most suitable to supervisees with various characteristics and nature [13], to emphasize that the main task of the supervisor is to follow the level of development and needs of the supervisees, then provide appropriate supervision environment, focuses and methods [12].

We tried to establish our supervisor-supervisee relation on the base of recognizing the characteristics and autonomy of social workers, working with the workers in the communities, understanding their problems through their perspectives, and discussing with them case by case instead of just giving suggestions as an outsider. In this vein, we could establish an equal relationship of cooperation to meet the social workers’ expectation and to practice the organization’s specialty and strength into real and usefulness.

Therefore, the first mission to start this study was to help these less-experienced workers face those key issues in community care: establish professional relation with the high-care elderly cases, lack of full resource network links that make them cannot implement their treatment plans without resources and cooperating supporting partners. As some social worker said in the first supervision meeting:

The conditions of these elders were mostly degraded, the situation was not easy to maintain, and the occurrence of illness or family accidents was always unpredictable. The treatment plan often faces changes, such as non-long-term follow-up cases, unless the staffs were familiar with the community and official resources network, or it is very difficult to fully grasp the case and change the situation. "........... the group supervision meeting record A01

Apparently, we have to consider that problem, so many social workers in the community care sites need to struggle for formal and informal resource and supports, since the operating structure could not be changed, the possible way to empower them and increase their quality of service should be focus on decreasing their professional burden and difficulties, and develop a possible case management model that is simple but clear and easy for using to help these workers to do their job better, easily, and quickly for saving these social worker’s time, energy and professional passion; then we shall expect the community care sites implement their real function to help the high-care elderly.

In addition, we still have to consider that there were various kinds of communities, types of high-care elderly and different sorts of needs high-care elderly clients that make these worker’s skills and knowledge from former common training cannot implement in the community care sites, the strategy of supervision have to be more flexible to be adopted in various situations in the communities, like quotes below:

“Now we social workers in the communities always face some main difficulties, like: (1) we introduced some unsuitable resources to clients or transferred them to useless network, resulted in resource waste or idle, because actually we didn’t have enough time before to figure out what they think about their problem and real needs; (2) since we social workers were often the only one professional worker in the site, so we have to depend on volunteers to visit our clients, but you knew there were a big gap of their service quality and professional level for each one of them; (3) the situation of those high-care elderly were always very complicated, so that make us hard to tell their type of demand” ........... the group supervision meeting record A04

LTC policy of Taiwan expects the community care site implement three of four main functions as mainstream primary care center in the community: “(1) usual visiting and telephone call caring, (2) consultation and referral services, (3) catering services and (4) health promotion activities”, since that we apparently have to consider not just needs of high-care elderly but also characteristics and special strength of these site that they could construct their own model to handle these challenges, as we quoted below, the resources that social workers between different positions could grasp were very different, this condition push us to have a flexible supervision strategy to adapted to characteristics and strength of these sites and to empower these workers to progress by their strength and what they still have, not what they cannot do or didn’t have.

“Because governmental subsidy was always very limited, apply qualification relatively has no elasticity, once the clients’ welfare identity changed, we have to find our own way to deal with that problem, like: to transfer to other NPOs or try to struggle resource from our own foundation, or coordinate with local community charity societies, a qualified social worker in the community has to share his/her resource network and experience to other workers nearby, and accumulate his/her own experience to get alone with bureaucratic system and rules, then gradually develop his/her expertise and service innovation. (Said by a relatively experienced social worker in community care site) ”…… group supervision meeting record A02

Through the discussion, we could understand that development of community care and high-care elderly case management model could not just rely on formal resources and the government, but also need to cooperate and coordinate with local community welfare units, to develop own unique social capital, then could implement every site’s characteristics and specialty, in this study, many our interviewees mentioned that many low service capacity sites may lack of these efforts.

So our supervision and strategies discussion must have stacked on characteristics of the community the clients, and the organizations, but also very important to show empathy to social worker’s limitation and difficulties to develop a balanced strategy to effectiveness and efficacy.

But this is a key problem for community social workers. Because although government’s budget cannot support all costs of the community care site, it still was the main target for a social worker in the site has to struggle for, no matter for passing the evaluation or struggling for community member’s recognition, the workers has to work hard to promote their strongholds, promotional activities and services, difficulties and troubles were everywhere but hard to speak out or record down, the social workers were forced to something that could earn more credit or budget but not actually those things could really develop their own strength and create their special future.

We confirmed the first principle of supervision of this case management model, focusing on examining the comparative advantages of various sites and their social workers. Based on this, we could make recommendations to overcome shortcomings, seek for empowerment through the strength perspective, and not to focus on weakness and regard it as challenges that have to be overcome in the future. The expecting progress will not be limited from other paradigms, exceeding one’s own characteristics, so the role of supervisor was no longer an authority to teach or control the social workers, but to work with them to discover their own strengths and specialties.

“A younger social worker said: it seems to me that by the case management model’s flow chart, it help us to classify different types of problems for high-care elderly, we less-experienced worker could use it to connect to resource network easily, help us to accumulate our experience before we could consider the process of intervention right and quickly. But we still have to create our own characteristics in our sites to improvement of service…after all, we were not like the other workers” …… group supervision meeting record A07

### Effectiveness of the strength perspective for community social workers

Function of social work supervisor is multiple, in addition to being a facilitative, counseling, and educational role for professional counseling, it is also a role for advocates and promoters who drive more professional services, also a supporting role for their workers to confirm career promise and recognition to profession. So, our supervision model didn’t judge social workers of the community care site, but appreciate their hard working and unique effort to implement their characteristics, then encourage them to use their strength to improve the service strategy and case management model.

For example, site J, with enough and stable social worker and volunteers human resources, their volunteers could visit the high-care elderly once every 1 to 2 weeks, the social worker regularly assess the welfare needs of clients, and intensively provide services such as welfare counseling and emotional support, but their cooperation between external resources and services was less, considered the site’s located in downtown that actually have many possible opportunities to cooperate with other NPOs, so our supervisors discussed with workers about the guideline of possible intervention to different types of high-care elderly by their strength:

“As for healthy aged clients, keep on monitoring their physical and psycho-socio condition, maybe we could cooperate with the neighbor NPO like “China Youth Corps “branch, daycare center or community development center nearby to encourage the elderly clients to participate our activities; about the volunteers, to establish regular training courses to keep quality of service stable. As for clients with depression, we can connect “TEACHER CHANG” FOUNDATION “Kaohsiung branch or mental health center of community that is near to our location to help providing mental consultation service that could integrate into our case management, increase interpersonal interaction and sense of self-identity. Finally, let’s review our intervention and plans together to discuss the effectiveness and challenges to decrease working burden and emotional burnout of workers and the organization.” ……supervision meeting record J101, J202, J301

In site H, it’s a branch of a national NPO for caring the elderly and promoting geriatric welfare, with rich internal resources and backup home care service resources, connection to external resources were also very completed, with sufficient social work manpower, allow the social workers could put more time for client’s care and case management, so our supervision strategy under the perspective of strength was like below:

“Perhaps we could cooperate to our original foundation and other activity center to broader the resource network connection, especially when we face clients with disabilities that already lost their daily living abilities, give a try to coordinate to our original organization to do more favors to family caregivers mental consultation, through our special relational network and caring secretary institution, introduce our assistive device service system and connect to formal and informal resource network to improve our service capacity to low socio-economic status and lonely dependant disable aged person. Especially when we have a experienced team and workers, have devoted deeply for a long time, we should empower our staff by each others’ expertise and support with each other, for example, organize a self-helping group to increase worker’s professional ability and recognition for the professional role and meaning of their job.” …… supervision meeting record J101, J201, J303

Another case worthy to mentioned is Site F, they located in a remote district near to rural mountain areas, rate of poor aged person was higher than other areas in Kaohsiung city, so they had the most high-care elderly cases with highest level of risks, has almost no external connection, but owned a very experienced social worker already worked there for a decades, and just lived in the community, deep dedication and rich sense of community consciousness was her unique and irreplaceable strength, so our supervision strategy focus on the social worker’s strength and utilize her advantages to expend the influence of the community care site.

From these cases we shared above, readers could understand that our idea is to focus on supervisee’s strength, rather than make them perfect, not value them by concept of cost–performance ratio, numbers of service user or any manageralism idea that only emphasize quantitative performance, but used user and individualized oriented perspective to discover and empower the community care sites and their social workers, by their own characteristics and strength. By analysis of our meeting record, many social workers expressed their appreciation to our supervision model that makes them feel supported and inspired, help them improve their quality of service and job loading, furthermore, they see changes of their organization, not to compare to other NPOs any longer, but compare to themselves and progress by themselves:

“Consultation with the specialist meant a lot to us, makes us could image new possibilities and progress by ourselves, so that we could create new service resource from new sources, even to establish evidence-based prove to improving quality of service. “……group supervision meeting record A04

Our supervision to the sites and social workers provide an opportunity for them to discuss their cases and share experience, difficulties, helplessness and imagination for the future of the organization to overcome the structural limitation that meant they were often have work and think alone.

“Before we joined this research project, my site always value client’s condition and needs only by my experience and knowledge, sometimes I felt confused or confident, but the fact was we have no extra resource so I have to struggle alone, by this supervision model and case management model, I knew more about the meaning of my judgment and evaluation, matched my experience and knowledge development, I even found that our effort is worthy to be appreciated, now I knew I’m a valuable important professional staff of community care system, and feel more familiar to the role I shall play”.……group supervision meeting record B02

Through this study, we developed a supervision model from the perspective of strength and applied it to clinical community care sites to help their social workers. Within the model, the social workers were provided opportunities to empower and re-interpret themselves, to re-establish their professional confidence and meaning of existence for their organization, and eventually to produce positive effects on quality of service and service receiver’s satisfaction.

## Discussion

### Social work supervision model and its’ effect under strengthen perspective

As we mentioned above, governmental budget supplement of human resource arrangement for community care site was very limited, so these community care sites have to be sustained by local NPO by their own funding and resources to meet the goal of welfare pluralism: inspire community consciousness, boost social service capacity, and decrease government’s social welfare budget loading. But it also resulted in unavoidable structural limitation for development of community care sites:

1. Because of geographic location and physical environment limitations, communities of remote areas often faced with dilemma of resources lacking that influence their normal function and human resource keeping, and even influence their model of operational management, decrease the possibility that allow the community care site operate sustainably, under such a vicious circle, experience of clinical service and human resource empowerment, operating data and files were not necessarily able to inherit, social workers may not have a stable and sustainable working environment to accumulate clinical experience and develop innovative service models, many communities that lack of funding and resources chose to hire part-time workers or even volunteers to provide borderline service reluctantly [14, 15].
2. For those NPO that come from other areas, once they found that operating these community care site may impact their original operating efficiency and return on investment, how they respect subjectivity for these community care sites of remote areas were always the debating issues, and influence practical function and residents’ perception for community care site [16]. These opinions were the reason that we tried to do this study to solve the problem, and we did see some positive influence in our samples.

We all knew that level of specialization and clinical experience of care service provider influence quality of care management mostly, but function of primary care service of community care site mostly were maintained by local part-time worker or unprofessional volunteers, these structural problems made these poor community care sites could not provide professional and qualified case management service, also meant, money and resources wasting [15], especially in remote areas, they didn’t even have enough functional and energetic volunteers, that made educational training courses for human resource empowerment of community care sites invalid [16]. Therefore there were some difficult situations for community care site to realize their primary care function, for example, their services could not help elderly with serious mental or physical problem, couldn’t solve or even ease social dilemma and risk of high-care elderly efficiently; because lack of professional human resources and funding, even they could provide various daily activities, but lack of useful courses such as prevention of disability and long-term care skills to achieve the goal of primary care for the elderly in the community. Generally speaking, Community care sites in Taiwan have problems that could not provide qualified, useful, stable and sustainable service, because of resource lacking and urban-rural gap.

Rapp and Goscha [17] pointed that the supervision model from the strength perspective emphasized on the reaction to pathological viewpoints at the theoretical level, initiated paradigm transfer, and not to worked in the problem oriented approach. The supervision model advocates the following viewpoints to promote changing:

1. The “problem” and “difficulty” are constructed and can be reconstructed.
2. To find flexible and diverse ways to make the work better.
3. The supervisee is an expert in his own situation. Worker-determination oriented approach is necessary.

Only combine the above points in the clinical application could really give full play to their own advantages and problem-solving strategies.

The supervision in this study was based on the above theoretical viewpoints and mechanism to strengthen those social workers in the community care sites, to make the workers being confident with their abilities to improve existed problems, to analyze the existed problems and limitations from different perspectives, to look for turning points or developmental opportunities by the existed advantages and resources but ignored in the past of the sites and the workers, to seek for external community resources that were not realized in the past, and to develop flexible and diversified challenge-overcoming strategies. In order to create individualized intervention methods for the social workers, to enhance communication and creative thinking through supervisory meetings, and to help workers have the autonomy to interpret and create new solutions, we could collect the response of unique growth experience and gains, seek alternative possibilities and effects of solving, and encourage social workers not to feel their own shortcomings from a particular paradigm model in the follow-up interview process.

### How the supervision model under strengthen perspective work?

However, situations of various organization reigned from different environments and social structures, the service needs and service volume of each community care site were impossible to be the same, so we decided to adopt the strengths perspective, to help our supervisees understand and utilize their own unique strength and resilience [18]. The strengths perspective regarded client’s weakness and low socio-economic status come from resource obtaining problem in their living situation, the reasons of their difficulties were actually because of they didn’t get chances and opportunities to empower and prove their strengths and potentials. Through this perspective, the key point of social work supervision and evaluation was no longer about how far they could reach the top or their weakness that need to be corrected, but admit the existence of various characters should be regarded as normal and reasonable things, and our strategy of supervision should focus on organization’s unique strengths and resilience, overcome problems and emphasize on possibilities, so supervisor’s focus should to help and encourage supervisees to discover their power and confidence to obtain necessary resources to utilize and develop their potential and intelligence [19–22]. Saleebey [23] pointed out that we could find source of the strength through three ways: individual resilience, membership, cultural approach, to participate in the community, to interact with members of community, create more strength and chance of innovation and cooperation, increase their ability to against social difficulties and oppressions, was a key to empower an organization or client to seek for strength and resilience.

From the perspective of clinical community geriatric social workers in Taiwan, they often face difficult situation in practice like: numerous cases that made workers over loading, scarce resources from the organization they were belong to, lacking of professional peer support system, weak employment protection with low working conditions, and these phenomenon made the community care site’s human resources were highly mobile and unstable, the incumbents have strong sense of helplessness, controlessness, low professional autonomy…etc., make us worried about these workers suffered from disempowerment and decreased their quality of service and caring functions [14]. But recent years, the effectiveness of supervision for easing disempowerment of clinical social workers, have been confirmed by many evidence-based researches and admitted by governmental authority, like nursing home accreditation institution [24–26], Pearson [27] and Shulman [28] also pointed out that positive and united supervisor-supervisee relationship would could bring professional growth to social workers and also increase their service effectiveness; these argument believe that a social worker with empowerment of supervision could transferred their professional growth into care and holistic care for clients, they could focus more on client’s strength, create efficient environment to help their client, so that the more effective the supervision as perceived by the social worker, the greater the client’s growth.

Those who gain the advantage form empowerment of supervision were confirmed that feel more confident, with more sense of responsibility, have tends to take action, cooperate and coordinate with the teamwork, with more ability to prevent from working burnout and mental exhaustion [26]. To those social workers in community care sites that often face the difficulties of over loading numerous cases but lack of support system, the key point that makes this supervision model of the strength perspective work was to let social workers have the autonomy to interpret the challenges they face and to think possible alternative solutions creatively, rather than being reviewed and blamed blindly for shortcomings and deficiencies [29] in order to meet the policy goal of long-term care in Taiwan: “Aging in place” by services with local characters.

We believe the supervision model of the strength perspective could be their important supporting and empowering resource. In research result of this study, we confirm that workers in the community care unit did feel supported and have more service capacity by using their own unique strength in the supervision model of strength perspective, confirm the views discussed in the above literature.

## Conclusion

### Meaning of case and care management under the perspective of strength

By the analysis of this study, we knew that it was not easy to run a community care site, especially lack of resources and manpower to maintain sustainable service, but the government gave these sites a role with heavy duty to practice primary care, so they also receive some inappropriate social reputation [5, 30, 31]. But in our researching cases, we used the perspective of strength to help them, to discover their own characteristics and unique strength develop their own resilience, to face oppression from the social and political structure and to create their only new strategy of service, to inspire social workers’ potential and confidence, so we generate our finding as below:

1. The perspective of strength focus on supervisee’s growth and resilience, matched the orientation and key concepts of case management: strengthen and optimization.
2. The perspective of strength focus on need oriented of supervisee rather than supervisor’s provision matched the ideal of case management: client-centered.
3. The perspective of strength emphasizes on take advantage of supervisee matched the ideal of case management: community-based.
4. The perspective of strength emphasizes on respect of supervisee’s characteristics and specialty, matched the ideal of case management: customized.
5. The perspective of strength argued that the supervisees have to create and activate their community support network, the idea is helpful to the concept that case management model valued: construction and enforcement of resource network.
6. The perspective of strength emphasizes on supporting and encouraging supervisee to increase their self-esteem and professional power, enhancing their cooperation to clients, partners, and environment that could reproduce their unique strength.

This study showed that create a supervisee-friendly psycho-socio environment empower these worker, by increasing their resource network and taking advantage of what they still have and what they are good at, this could help these worker upgrade their level on ladders of empowerment, not as a staff that only could be manipulated or be told, but become an active and independent professional worker, that have power and confidence to make treatment and intervention.

### The importance of professional manpower and their empowerment in the community care sites

According to our research result, we found that professional human resource is very important to the quality of service and the effectiveness of case and care management model, empowerment could help them upgrade their professional confidence and broader their network connection, improve quality of comprehensive geriatric assessment and intervention implement for high-care elderly clients. So we strongly recommended that all community care site should hire at least one qualified full-time social worker to devote into the community that could really improve quality of service, and provide necessary supervision and empowerment resource to maintain their service and care capacity.

### Enforce cooperation and coordination between community organizations and connect their resource network

By our research result, most social workers agreed that to communicate and share their experience and resource connection network, was very useful and inspiring. So we suggest organizers of the community care sites should enforce cooperation and coordination between community organizations and connect their resource network regularly.

### Establish a supervision model under the perspective of strength, individualization, and interdisciplinary cooperation

Social work is a career that essentially a highly interdependent collective team work, provide supervision supporting and empowerment resources, could keep and even increase worker’s expertise, career promise and quality of service upgrade, it’s necessary for welfare of the elderly but also for worker’s labor right and well-being.

So we suggest all community care sites should provide supervision programs under the perspective of strength, individualization, and interdisciplinary cooperation for their staff, to strengthen their professional support, and stabilize their condition of human resource training, keeping and utilization.

## Data Availability

The data used to support the findings of this study have not been made available because of the reason for the protection of privacy and ethical right of our research participants. In case anyone may need to access the data, please contact to the corresponding author Professor Kuei-Min Chen, PhD, RN, FAAN, Kaohsiung Medical University College of Nursing, and Kaohsiung Medical University Hospital Department of Medical Research,100Shih-Chuan1stRd., Sanmin District, Kaohsiung, Taiwan 80708 (e-mail: kmc@kmu.edu.tw).
